# Comparative effects of intensive ganglionated plexus ablation in treating paroxysmal atrial fibrillation and vasovagal syncope

**DOI:** 10.1002/clc.23446

**Published:** 2020-08-17

**Authors:** Xingfu Huang, Yanjia Chen, Yuli Huang, Hongxin Zhao, Liwei He, Zhenni Tan, Dingli Xu, Jian Peng

**Affiliations:** ^1^ Department of Cardiology, Nanfang Hospital Southern Medical University Guangzhou China; ^2^ Department of Anesthesiology, Nanfang Hospital Southern Medical University Guangzhou China; ^3^ Department of Cardiology The First People's Hospital of Shunde Foshan China; ^4^ Shanghai Synyi Medical Technology Co., Ltd Shanghai China

**Keywords:** atrial fibrillation, catheter ablation, ganglionated plexus, vasovagal response, vasovagal syncope, pulmonary vein isolation

## Abstract

**Background:**

Ganglionated plexus (GP) ablation is used to treat atrial fibrillation (AF) and vasovagal syncope (VVS). However, the comparative effects of GP ablation in treating paroxysmal atrial fibrillation (PAF) and VVS have not been well studied.

**Objective:**

The purpose of this study was to investigate the effects of intensive GP ablation on PAF and VVS.

**Methods:**

PAF and VVS patients were enrolled in this study. Pulmonary vein isolation (PVI) was performed in the PAF group, and additional ablation was performed at GP sites. Anatomic ablation of left atrial GPs was performed in the VVS group. The primary endpoint was freedom from AF or other sustained atrial tachycardia and syncope recurrence.

**Results:**

A total of 195 patients were enrolled: 146 patients with PAF, including eight patients with combined VVS (PAF group), and 49 patients with VVS (VVS group). Vasovagal response (VR) was achieved in 78 (53.4%) patients in the PAF group and 48 patients (98.0%) in the VVS group (*P* < .05). During the 17.8 ± 10.5 (range, 3‐42) month follow‐up, 126 (86.3%) patients were free of AF in the PAF group, and 45 (91.8%) patients in the VVS group had no syncope recurrence and significantly improved symptoms.

**Conclusions:**

Anatomically guided intensive GP ablation showed efficient clinical outcomes for both groups of patients. Compared with PAF patients, VVS patients had more VR during ablation in the left atrium. Furthermore, VR during ablation indicated a better prognosis in PAF patients.

## 1 INTRODUCTION

Numerous conglomerations of autonomic ganglia are present on the epicardial fat pads of the heart and are known as the ganglionated plexi (GPs).^1^ These GPs play an important role in the genesis of atrial fibrillation (AF),^2^ symptomatic bradycardia, and vasovagal syncope (VVS).^3^, ^4^ Four major GP sites are distributed in the left atrium and have been widely reported.^4^, ^5^, ^6^ GP ablation usually leads to a vasovagal response (VR) and is used to treat VVS^1^; this treatment has been widely studied, and convincing results have been reported.^3^ However, ablation of the right anterior ganglionated plexus (RAGP) usually leads to an increased heart rate (HR),^7^, ^8^ another reaction of VR. Furthermore, targeted GP ablation in addition to pulmonary vein isolation (PVI) improves the outcomes of paroxysmal atrial fibrillation (PAF).^2^, ^5^, ^6^, ^9^, ^10^, ^11^ In fact, VR during ablation is different between AF and non‐AF patients,^12^ and these responses are also an independent risk factor for AF recurrence after radiofrequency (RF) or cryoballoon ablation,^6^, ^11^, ^13^ but the GP ablation response relationship between PAF and VVS patients remains unknown.

The aim of this study was to identify the distribution of GPs in PAF and VVS patients. In addition, the comparative effects of intensive GP ablation were compared between the two groups according to the anatomic GP location.

## 2 METHODS

### 2.1 Patients

We prospectively studied (between April 2016 and September 2019) consecutive PAF patients referred for catheter ablation. Each patient was ≥18 years of age and had symptomatic PAF despite the use of one or more class I or III antiarrhythmic drugs. Patients with persistent AF were excluded, and additional exclusion criteria included left ventricular ejection fraction (LVEF) <35%, left atrial diameter (LAD) >50 mm, New York Heart Association class III or IV heart failure, and a previous history of heart surgery or catheter ablation.

Consecutive patients with frequently recurring episodes of VVS were enrolled in the VVS group. All patients had a history of more than three syncopal episodes or at least one recurrence of syncope within 6 months before catheter ablation and a positive response in head‐up tilt testing (HUT). All patients experienced failure of conventional treatments, including optimal fluid intake, physical counterpressure training, and pharmacological treatments. Permanent pacemaker implantation also failed in two patients. Treatment failure was defined as the spontaneous occurrence of syncope. Previous medications were all discontinued before ablation. Other causes of syncope, including sinus node and atrioventricular (AV) block, hypertrophic cardiomyopathy, transient ischemic attacks, subclavian steal syndrome, and drug‐induced syncope, were excluded. Additional exclusion criteria were as above but for PAF patients.

Clinical histories were obtained from all patients, and baseline assessments were performed before the procedure and included 12‐lead ECG, 24‐hour ambulatory monitoring (Holter), and echocardiography. A thrombus in the left atrial (LA) appendage was excluded in all PAF patients before the procedure. The local research ethics committee approved the study, and written informed consent was obtained from each participant upon enrollment.

### 2.2 HUT‐testing

The detailed HUT protocol was described by Yao.^14^ Patients were first tilted at 70° for 30 minutes. If no symptoms occurred, the participants were treated with 0.25 mg of nitroglycerin (NTG) sublingually and continued to be tilted for an additional 20 minutes (NTG tilt testing). A positive response to HUT was defined as cardioinhibitory when a marked bradycardia (≤40 bpm for >10 seconds) or prolonged asystole (>3 seconds) occurred at the time of syncope. A positive response was defined as vasodepressor or mixed when isolated hypotension or hypotension associated with only mild bradycardia (≤40 bpm) or a brief asystole (>3 seconds) was observed.^15^

### 2.3 Electrophysiological study, mapping, and ablation

All antiarrhythmic drugs were discontinued for at least five half‐lives before the procedure. All procedures were performed under conscious sedation with intravenous administration of fentanyl and midazolam for analgesia. Pulse oximetry and blood pressure were monitored during the procedure.

Femoral veins were punctured. A 6F decapolar steerable electrode catheter was placed in the coronary sinus, and a 6F quadripolar electrode catheter was positioned in the right ventricle apex. A routine conventional electrophysiological study was performed on all patients before ablation. LA transseptal puncture and three‐dimensional electroanatomic mapping were performed as in our previous studies.^16^, ^17^

In the PAF group, PVI was performed as previously described.^16^, ^17^ The PVI circumferential lines were designed at a distance of 0.5 to 1.5 cm from the ostia of the left and right pulmonary veins (PVs). Four major GP sites are located in the left atrium, which are empirically identified as follows^3^, ^4^, ^10^, ^18^, ^19^: (1) left superior (LS) GP (usually located in the superolateral area around the root of the LSPV or between the anterior ridge and LSPV); (2), left inferior (LI) GP (located in the inferoposterior area around the root of the LIPV ostium); (3), right inferior (RI) GP (located in the inferoposterior area around the root of the RIPV); and (4), right anterior (RA) GP (anterior aspect of the right PV vestibulum). RF ablation was delivered using an ablation catheter (Thermocool SmartTouch [ST] or Thermocool SmartTouch Surround Flow [STSF], Biosense Webster, Inc. Diamond Bar, California) using a contact force of 5 to 20 g with a time of 20 to 30 seconds, and the ablation power was maintained at 30 to 35 W. The order of PVI proceeded from the left to right circle. During PVI, care was taken to monitor the reaction to GP ablation, including the VR, which was defined as transient ventricular asystole, AV blocking, an increase in the basal cycle length (BCL) interval of 50%,^19^ or an increase in HR when ablating the RAGP, defined as a 20% increase in the BCL. Thus, extra‐ablations were performed at the anatomic sites of the GPs until the VR disappeared. The endpoint of PVI was the verification of the PV potential by demonstrating both entrance and exit block with the use of a PentaRay mapping catheter (Biosense Webster, Inc. Diamond Bar, California). Furthermore, AF trigger was also mapped, and ablation, such as superior vena cava isolation or additional lines of ablation, was performed according to each patient's condition.

An electrophysiological study was consistently recorded before ablation. The BCL, Wenckebach cycle (WC) length of the AV node and corrected sinus node recovery time (CSNRT) were measured. Anatomically guided GP ablation was performed in the VVS group. The order of ablation was preset as follows: LSGP‐LIGP‐RIGP‐RAGP. VR at all GP sites was observed during ablation.^19^ At each GP site, if the tentative delivery of RF energy induced any VR‐response within 10 seconds, then additional energy was delivered for 20 to 30 seconds, and right ventricular pacing was applied if necessary. Intensive ablation was performed around the site until VR was inhibited. If no VR appeared at the presumed GP site, additional ablation was also performed at a minimum of 4 to 6 points for 20 to 30 seconds at each site. The procedural endpoint was defined as inhibition of VR at the target sites.

After ablation, BCL, WC length, and CSNRT were measured to evaluate the ablation efficiency. Intracardiac electrograms were filtered from 30 to 500 Hz and measured at a sweep speed of 100 mm/s.

### 2.4 Postablation follow‐up

After the procedure, all patients were observed as inpatients for at least 1 day. The PAF group was prescribed amiodarone and rivaroxaban or dabigatran for at least 2 months. The VVS group was given metoprolol and aspirin/rivaroxaban for at least 2 weeks. The postablation follow‐up consisted of a clinical visit at 3 and 12 months after ablation. Furthermore, the patients could also contact medical personnel via phone or computer‐based instant messaging. Recurrent symptoms or syncope were carefully monitored. The primary study endpoint with respect to ablation efficacy was freedom from AF or other sustained (duration >30 seconds) atrial tachyarrhythmia (AT) using a Holter monitor or syncope.

### 2.5 Statistical analysis

All data are reported as the mean ± SD for continuous variables and as the number of subjects (%) for categorical variables. Independent sample *t*‐tests were used to analyze differences between the two groups. Paired *t*‐tests were used for comparisons of pre‐ and postablation measures. Categorical variables were compared using Pearson χ2 analysis or Fisher's exact test. The time to AF recurrence was estimated by the Kaplan‐Meier method, and comparisons were made with the log‐rank test. Univariate and multivariate Cox proportional hazards models were used to identify significant predictors of recurrence events. A two‐sided *P* value <.05 indicated statistical significance. Data were analyzed using the SPSS statistical package for Windows, version 20.0 (IBM Corp., Armonk, NewYork).

## 3 RESULTS

### 3.1 Patient characteristics

In total, 195 patients were enrolled in this study: 146 patients with PAF, including eight patients with combined VVS (PAF group), and 49 patients with VVS (VVS group). Detailed clinical data of the enrolled participants are listed in Table 1. All VVS patients were HUT‐positive, and six patients had physical injury caused by syncope. The patients in the PAF group were older, had a faster HR, a larger LAD and a lower LVEF than patients in the VVS group (*P* < .05). However, the VVS group had more females and a longer disease history (*P* < .05).

**TABLE 1:** Baseline characteristics of study patients between the two groups

	PAF group (n = 146)	VVS group (n = 49)
Age, years	61.1 ± 10.5	42.4 ± 16.1^a^
Gender, male/female	92/54	22/27^a^
Heart rate (bmp)	71.3 ± 9.2	65.4 ± 7.3^a^
Body mass index (kg/m2)	23.5 ± 3.9	23.1 ± 3.2
History, months	19.9 ± 15.9	39.8 ± 39.4^a^
Left atrial diameter (mm)	38.4 ± 4.0	35.4 ± 2.9^a^
Left ventricular ejection fraction (%)	60.0 ± 6.9	62.9 ± 6.2^a^
VVS types	VVS in PAF (n = 8)	–
Cardioinhibitory type	6	14
Vasodepressor type	0	6
Mixed type	2	29

*Note*: Values are presented as mean ± SD or as n (%).

Abbreviations: PAF, paroxysmal atrial fibrillation; VVS, vasovagal syncope.

^a^ *P*‐value <.05 was considered significant.

### 3.2 Procedure parameters

The total durations of the procedure were 155.3 ± 9.5 and 97.6 ± 10.9 minutes, and the ablation times were 92.4 ± 11.3 and 13.5 ± 2.8 minutes in the PAF and VVS groups, respectively (*P* < .05). Three tamponade complications occurred in the PAF group when LA transseptal puncture was performed, and one patient was referred for cardiac surgical repair.

**FIGURE 1:**
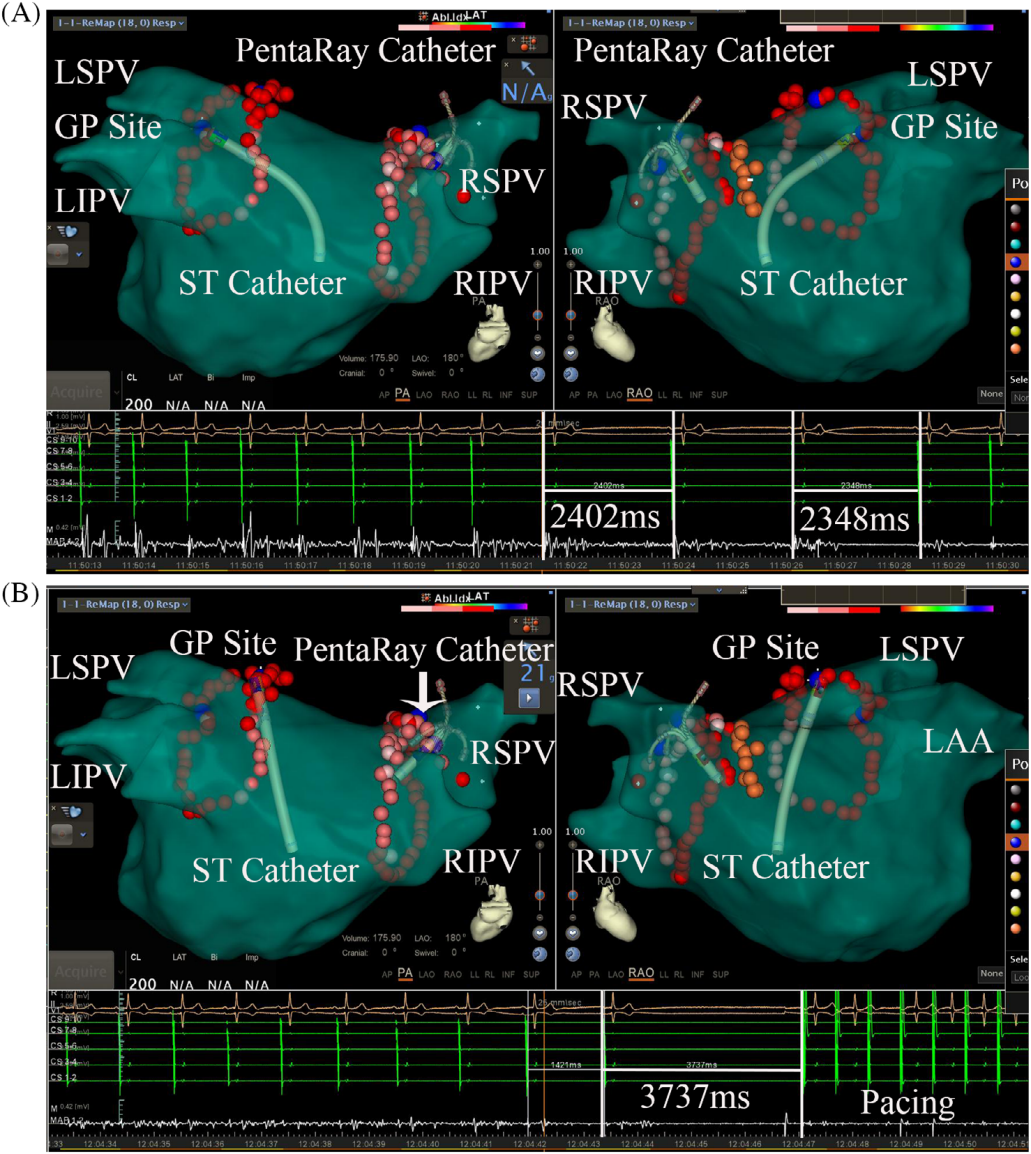
A and B shows a VR induced by delivery of RF energy during PVI in a 65‐year‐old patient. A geometric representation of the left atrium was constructed using the Carto 3 mapping system. The blue spheres represent a notable positive VR at each GP site. A, Shows a VR located between the anterior ridge and the LSPV. B, Shows a VR located in the superolateral area around the root of the LSPV. The red dots represent the ablation points on the LA geometric image. LAA, left auricular appendage; LIPV, left inferior pulmonary vein; LSGP, left superior ganglionated plexus; LSPV, left superior pulmonary vein; RIPV, right inferior pulmonary vein; RSPV, right superior pulmonary vein

During PVI in the PAF group, VR was achieved in 78 (53.4%) patients. The average number of GPs with a positive response was 2.1 ± 1.1 per person with a total area of 1.4 ± 0.5 cm^2^ (Table 2). Fifty‐six patients showed a VR at the LSGP, 28 of whom had responses in the superolateral area around the root of the LSPV; another 17 patients had responses in the anterior ridge and LSPV ostium, and 11‐patients had responses at both sites. Ablation as well as PVI were performed at GP sites. Figure 1A,B show a 65‐year patient who had a VR at both sites of the LSGP. In addition, 23 and 15 VRs were observed at the LIGP and RIGP, respectively. When ablation was performed at the RAGP, VR was shown in 68 (87.2%) patients. Compared to baseline, the BCL, WC length, and CSNRT were decreased after the procedure (Table 2), and the positive VR group had more significant changes in BCL and WC length decrease than the negative group (*P* < .05).

**TABLE 2:** Comparison of procedure parameters change among the three groups

	Positive VR in PAF group (n = 78)	Negative VR in PAF group(n = 68)	VVS group (n = 49)
GP response	78 (100%)	0	48 (98.0%)
VR	56 (71.8%)	0	47 (95.9%)^a^
RAGP	68 (87.2%)	0	45 (91.8%)
LSGP	56(71.8%)	0	47(95.9%)^a^
LIGP	23(29.5%)	0	30(61.2%)^a^
RIGP	15(19.2%)	0	21(42.9%)^a^
Average number of GPs	2.1 ± 1.1	0	2.9 ± 0.9^a^
Total areas of GPs	1.4 ± 0.5	0	2.7 ± 0.8^a^
BCL before ablation	843.6 ± 138.5	838.0 ± 147.0	918.4 ± 93.7^a^
BCL after ablation	713.0 ± 96.9	742.0 ± 95.4	681.6 ± 91.2^a^
BCL decrease %	14.5 ± 10.0	10.4 ± 8.7^b^	25.5 ± 9.6^a^
CSNRT before ablation	521.0 ± 173.9	525.2 ± 198.0	548.8 ± 192.9
CSNRT after ablation	370.1 ± 96.9	408.7 ± 151.7	322.5 ± 71.1^a^
CSNRT decrease %	25.9 ± 14.8	21.5 ± 12.9	36.2 ± 17.5^a^
WC length before ablation	434.2 ± 41.9	424.1 ± 40.9	464.5 ± 38.9^a^
WC length after ablation	373.7 ± 28.8	377.2 ± 28.7	344.5 ± 28.0^a^
WC length decrease %	13.7 ± 4.8	10.8 ± 4.2^b^	25.6 ± 6.0^a^
Freedom of events	72(92.3%)	54(79.4%)^b^	45(91.8%)

*Note*: P‐value < .05 was considered significant; values are presented as mean ± SD.

Abbreviation: BCL, basal cycle length interval; CSNRT, corrected sinus node recovery time; GP, ganglionated plexus; PAF, paroxysmal atrial fibrillation; VR, presence of vasovagal response; WC, Wenckebach cycle.

^a^ means significant different between VVS and positive or negative VR in PAF group.

^b^ means significant different between positive and negative VR in PAF group.

**FIGURE 2:**
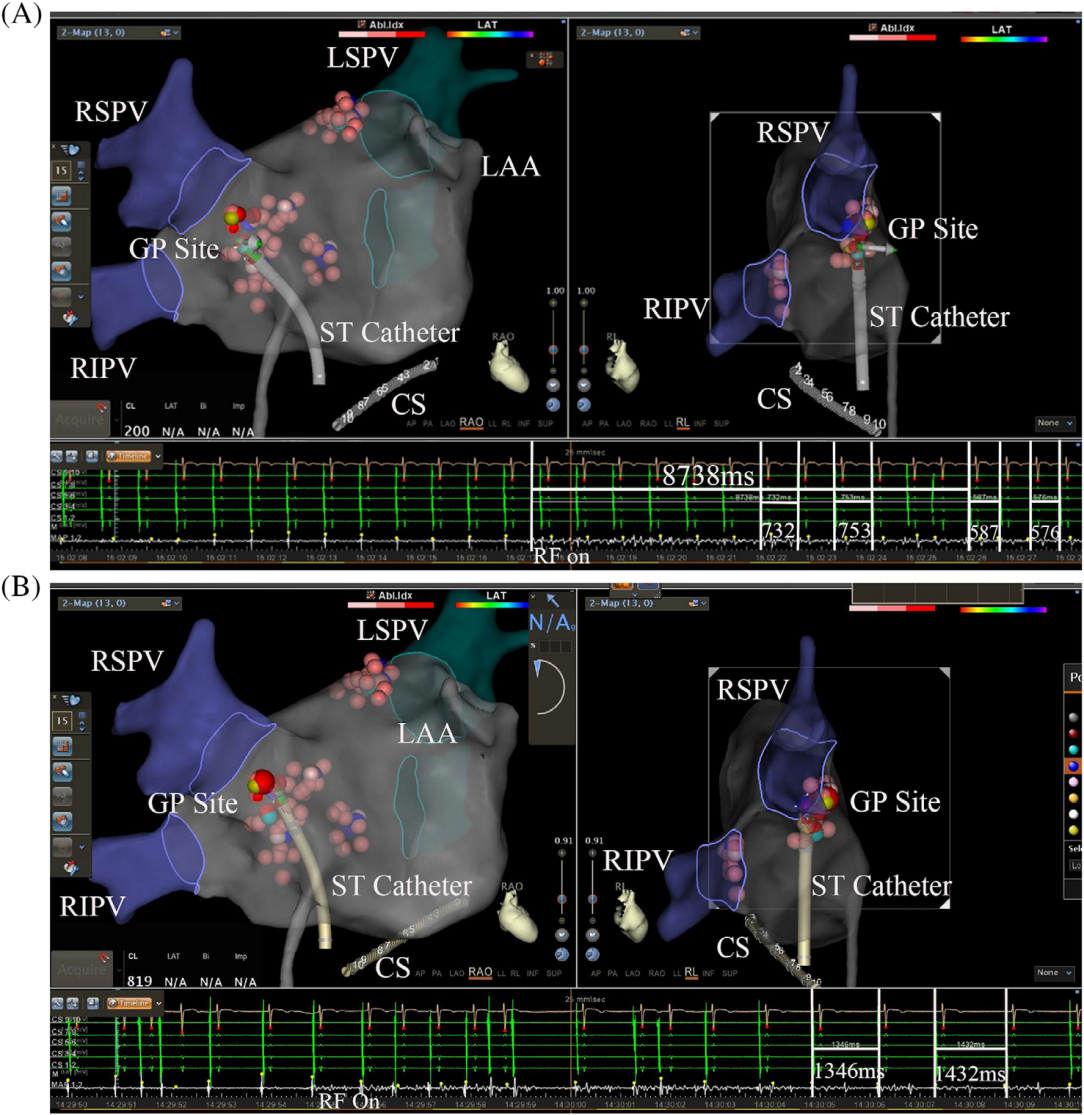
A and B shows a VR induced by delivery of RF energy at the RAGP in a 35‐year‐old man. A, Shows the RF energy delivered to the RAGP, and the R‐R interval decreased to 587 ms during the 8738‐ms RF time. B, Shows that RF was also delivered near the above site, but the R‐R interval increased to 1432 ms during ablation. GP, ganglionated plexus; LAA, left auricular appendage; LSPV, left superior pulmonary vein; RIPV, right inferior pulmonary vein; RSPV, right superior pulmonary vein

VR was achieved in 48 (98.0%) patients in the VVS group under anatomically guided ablation. The only patient who did not show a VR‐response had the vasodepressor type of VVS. VR was elicited at the LSGP in 47 patients (95.9%), and all responses were located in the superolateral area around the root of the LSPV; only 16 patients also showed a VR at the anterior ridge. Furthermore, a VR was elicited at the RAGP in 45 (91.8%) patients, the LIGP in 30 (61.2%) patients, and the RIPV in 21 (42.9%) patients. The average number of GPs was 2.9 ± 0.9 per person, and the average area was 2.7 ± 0.8 cm^2^ (Table 2). Figures 2A,B show an example of a positive VR as observed at the RAGP in a 35‐year‐old man. A significant and immediate shortening of the BCL, WC length and CSNRT was observed after ablation (Table 2). Compared with the positive or negative VR in the PAF group, VR, the number and area of the GP, the change in BCL, WC length and CSNRT were significantly different (*P* < .05), except the baseline of CSNRT before ablation.

### 3.3 Follow‐up

During a mean follow‐up time of 17.8 ± 10.5 (range, 3‐42) months, 126 (86.3%) patients were free from AF in the PAF group, including 72 patients in the positive VR group and 54 patients in the negative group (*P* < .05). More importantly, the eight AF patients with combined VVS were all positive for VR, and no one presented AF or syncope recurrence.

**FIGURE 3:**
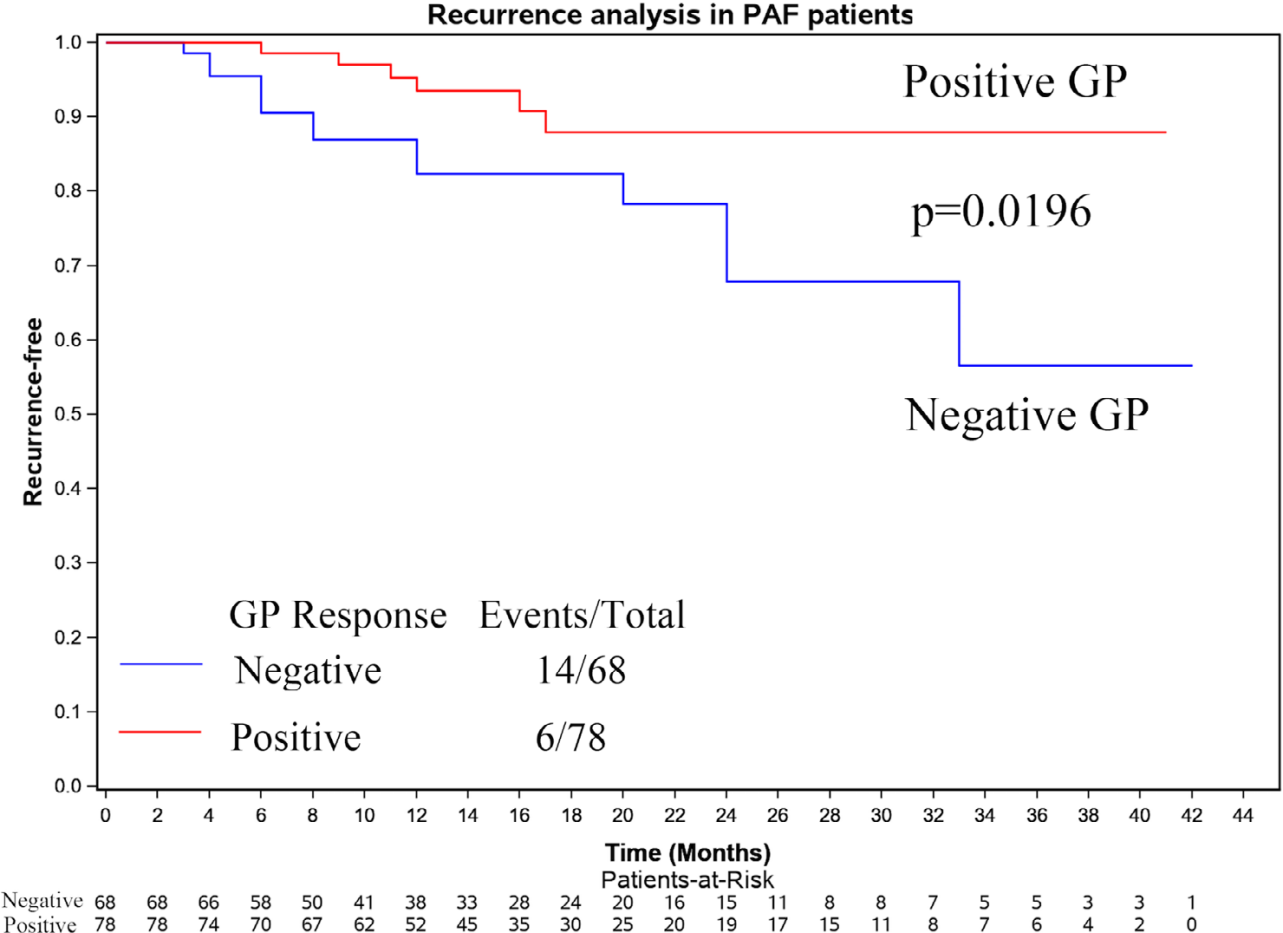
Kaplan‐Meier curve analysis of the mid‐term outcome of those with positive and negative GP responses in PAF patients (*P* = .0196). GP, ganglionated plexus; PAF, paroxysmal atrial fibrillation

The Kaplan‐Meier curve comparing the cumulative freedom from atrial arrhythmia recurrence is presented in Figure 3. Variables were fitted by a univariate and multivariate Cox model to assess their prognostic role regarding atrial arrhythmia recurrence during long‐term follow‐up. VR and total GPs were the most important risk factors for recurrence‐free survival. Furthermore, decreases in BCL and WC length were also significantly associated with the endpoint of recurrence‐free survival in univariate analysis but not in multivariate analysis (Supplementary Tables 1 and 2).

During a mean follow‐up time of 16.4 ± 9.9 months (range, 3‐42 months), all VVS patients underwent a clinical visit; 45 patients (91.8%) had no syncope recurrence. The remaining four patients (two with mixed and two with vasodepressor type VVS) had syncope recurrence, but the frequency of syncope was significantly decreased.

## 4 DISCUSSION

### 4.1 Major findings

This study revealed the following findings: (1) Anatomically guided intensive GP ablation was effective for PAF and VVS treatment, and a positive VR indicated a better prognosis in the PAF group. (2) The VVS group had a larger GP area in the left atrium than the PAF group, and VR was more significant in the VVS patients than in the PAF patients.

### 4.2 GP distribution in the PAF and VVS groups

The autonomic nervous system (ANS) has been shown to play an important role in the pathophysiology of AF.^2^ Intrinsic cardiac ANS, mostly formed by interconnected clusters of epicardial autonomic GP, is critical for both the initiation and maintenance of AF or VVS.^2^, ^3^, ^4^ Two methods previously existed to recognize the GP, high‐frequency stimulation (HFS) and anatomical location guidance, and the latter method may be superior.^20^ Therefore, the GP distribution was evaluated by the anatomically guided ablation strategy.

Our study revealed the GP distribution in different patients. A VR was achieved in 98% of VVS patients and in 53.4% of PAF patients. The rate was similar to that of previous studies.^6^, ^20^ These results indicate that GPs are more critical for VVS than PAF. Furthermore, the number and area of GPs were larger in VVS patients. Both LSPG and RAGP were the most common GP sites.^4^ However, the area of the GPs was smaller than that in previous studies.^2^, ^4^ This could have occurred because RF ablation may decrease the GP area, similar to the effect of cryoballoon ablation.^4^

In this study, we also found that VR was different at RAGP than at other sites. In fact, the RAGP response included an increase in HR. Hu et al. reported that ablation of the RAGP could increase the HR immediately and for the long‐term; it could also inhibit VVS occurrence.^7^, ^8^

### 4.3 Rationale for consolidated catheter ablation for PAF and VVS

In our study, PVI was a critical method for the treatment of PAF, and ablation of more lesions was performed if VR occurred. During follow‐up, 92.3% of patients in the VR‐positive group and 79.4% in the negative group were free from AF/AT (*P* < .05). This was similar to the report by Te et al.^5^ This finding may be explained by the following. First, Katritsis et al. reported that GP ablation with PVI confers a significantly higher success rate than either PVI or GP ablation alone in patients with PAF.^8^ Pokushalov et al. reported that PVI + GP ablation confers superior clinical results and reduced AF recurrence compared to PVI + line ablation at 3 years of follow‐up.^18^ Recently, a meta‐analysis also confirmed this conclusion.^13^ Therefore, intensive ablation at the GP site helps improve the prognosis. Second, Baykaner et al. detected focal and rotational sources of AF in the left atrium that were often colocalized with regions of the GP, and consolidated ablation can destroy AF triggers. Finally, negative VR PAF patients might be another trigger for AF, so it was different from mapping and ablation.

Endocardial GP ablation has increasingly been used to treat VVS, and many convincing results have been reported.^3^, ^14^, ^20^, ^21^, ^22^, ^23^, ^24^ The cardioinhibitory type of VVS is the most common type reported to be subjected to ablation^20^, ^25^; however, the mixed and vasodepressor types of VVS were also ablated in this study. The results indicated that GP ablation was effective for all types of VVS. Compared to GP ablation for the cardioinhibitory type, this treatment for the mixed and vasodepressor types of VVS demonstrated poor effectiveness.

The average number of GPs with a positive VR was 2.9 ± 0.9 per person in our study, which was higher than previously reported^20^, ^26^ and may have been due to the use of intensive ablation. The ablation number and time were much higher in our study, which led to larger ablation lesions. In addition, a saline‐irrigated catheter was used, which could lead to deeper lesions. Previous studies have indicated that catheter ablation was effective for VVS, but syncope sometimes reoccurred. Late vagal tonus recovery after denervation with various techniques is an important, well‐demonstrated issue.^14^, ^22^ Therefore, a significant portion of vagal innervation after the induction of RF lesions may be stunted but remain functional, and consolidated ablation might be useful. Considering that autonomic GP denervation potentially recovers long after catheter ablation, we elected to adopt an extensive LA denervation protocol in our study in addition to using saline‐irrigated catheters in a substantial number of RF ablations (more than four points at each GP location), which might be critical for achieving vagal tonus attenuation.

### 4.4 VR and prognosis of PAF and VVS patients

GPs have been reported to regulate sinoatrial and AV node function by interconnecting nerve fibers, thus affecting the intrinsic HR and conduction system.^27^ Efferent vagal inputs to the heart, which often cause bradycardia and AV conduction block, can be effectively inactivated by RF ablation of the GP. Clinical studies have shown that electrophysiological parameters are altered during ablation.^3^, ^20^, ^25^ We also observed a decrease in the BCL, CSNRT, and WC length after ablation, which was more significant in the VVS group and the positive VR group. Positive VR indicated a better prognosis in PAF patients, similar to previous reports.^6^, ^11^, ^13^ Furthermore, history, BCL, and WC length decrease were also significant in the positive VR group. Based on the above description, intensive RF ablation was performed until the VR disappeared.

### 4.5 Study limitations

This was a single‐center study that included PAF and VVS patients. Because of this design, the study patients were not fully matched. However, the GP distribution was found to be different between the two groups. Second, the VR in the PAF group was found based on the ablation lines. Intensive ablation was omitted if GP sites were located beside the ablation lines. Furthermore, the true anatomic location of GPs is epicardial, and thus, it is not always feasible to effectively abolish all GPs. In addition, GP ablation was effective in the study patients for a medium‐term follow‐up period, but long‐term follow‐up data on this procedure are still lacking.

## 5 CONCLUSIONS

Anatomically guided intensive GP ablation was efficient and feasible for PAF and VVS. The VVS group had a larger GP area in the left atrium and more VR during ablation than PAF patients. Furthermore, positive VR indicated a better prognosis in PAF patients.

## CONFLICT OF INTEREST

The authors declare no potential conflicts of interest.

## DATA AVAILABILITY STATEMENTS

The data underlying this article will be shared on reasonable request to the corresponding author.

## Supporting information


**Supplementary Table 1** Evaluating influencing factors on recurrence by univariate and multivariate cox

**Supplementary Table 2** Evaluating influencing factors on recurrence by univariate and multivariate cox
