# Supplementary material for: Comparative effects of intensive ganglionated plexus ablation in treating paroxysmal atrial fibrillation and vasovagal syncope
Source: Clin Cardiol. 2020 Aug 17;43(11):1326–33. doi: 10.1002/clc.23446 (PMC7661657; doi:10.1002/clc.23446)
Supplement: Supplementary file 1 — Supplementary Table 1 Evaluating influencing factors on recurrence by univariate and multivariate cox [file CLC-43-1326-s001.docx]

|  | Univariate analysis  HR(95%CI) | p-value | Multivariate analysis  HR(95%CI) | p-value |
| --- | --- | --- | --- | --- |
| Age | 1.03(0.98-1.07) | 0.29 | 1.02(0.97-1.07) | 0.38 |
| Sex (Female) | 1.42(0.58-3.51) | 0.44 | 1.19(0.46-3.12) | 0.72 |
| VR | 0.34(0.13-0.89) | 0.03 | 0.24(0.07-0.75) | 0.01 |
| AF history | 1.02(1.00-1.04) | 0.10 | 1.03(1.00-1.06) | 0.03 |
| BCL decrease | <0.01(0.00-0.59) | <0.01 | 0.01(0-3.96) | 0.13 |
| WC decrease | <0.01(0.00-0.11) | <0.01 | <0.01(0-254.3) | 0.32 |
| CSNRT descrease | 5.24(0.19-143.5) | 0.33 | 29.7(0.45-1975.1) | 0.11 |

**Supplementary Table 1** Evaluating Influencing Factors on recurrence by Univariate and Multivariate Cox

Regression Model in PAF patients (VR)
